# Muscle activity and rehabilitation in spinal stenosis (MARSS) after conservative therapy and surgical decompression with or without fusion: Protocol for a partially randomized patient preference trial on rehabilitation timing

**DOI:** 10.1016/j.conctc.2024.101273

**Published:** 2024-02-22

**Authors:** Eduard Kurz, Philipp Schenk, Florian Brakopp, Moritz Diers, Oliver Klingel, Stefan Bone, Hans Jörg Meisel, Karl-Stefan Delank, Bernhard W. Ullrich

**Affiliations:** a Department of Orthopedic and Trauma Surgery, Martin Luther University Halle-Wittenberg, Ernst-Grube-Str. 40, 06120, Halle (Saale), Germany; b Department of Science, Research and Education, BG Klinikum Bergmannstrost Halle gGmbH, Merseburger Str. 165, 06112, Halle (Saale), Germany; c Department of Trauma and Reconstructive Surgery, BG Klinikum Bergmannstrost Halle gGmbH, Merseburger Str. 165, 06112, Halle (Saale), Germany; d Saline Rehabilitationsklinik, Mansfelder Str. 52, 06108, Halle (Saale), Germany; e Department of Neurosurgery, BG Klinikum Bergmannstrost Halle gGmbH, Merseburger Str. 165, 06112, Halle (Saale), Germany

**Keywords:** Spinal stenosis, Decompression, Fusion, Surgery, Rehabilitation, Muscle function, Surface EMG

## Abstract

**Background:**

Patients affected by lumbar spinal stenosis (LSS) suffer from a multifactorial degeneration of the lumbar spine resulting in narrowing of the neuroforamina and spinal canal, leading to various functional limitations. It remains unclear whether LSS patients after surgery would benefit from early post-operative rehabilitation, or if a delayed rehabilitation would be more advantageous. The purpose of this partially randomized patient preference trial is to evaluate the impact of post-operative rehabilitation timing as well as surgical intervention type on psychometric properties and functional outcomes in patients with LSS.

**Methods:**

Data for this patient preference trial are collected before and after surgical (decompression only or decompression and fusion) and rehabilitative interventions as well as six, 12 and 24 months after completing rehabilitation. The study participants are patients diagnosed with LSS who are at least 18 years old. After a medical check-up, participants will complete patient-reported outcome measures (PAREMO-20, SIBAR, FREM-8, SF-12, SFI, ODI) and different functional assessments (functional reach test, loaded reach test, handgrip strength, standing balance control, 6-min walk test).

**Ethics and dissemination:**

The results of this study will be published through peer-reviewed publications and scientific contributions at national and international conferences. This research has been approved by the Institutional Review Board of Martin Luther University Halle-Wittenberg (reference number: 2022-128).

## Teaser

Strengths and limitations of this study.

• Patients' thoughts on early or delayed post-operative intervention and thus individual preference are considered.
• Information about the individual surgical procedure will be documented.
• Patients are recruited and treated in two different and independent spinal surgery centers.

## 1 Background

Lumbar spinal stenosis (LSS) is a common source of lower-quarter musculoskeletal pain resulting in meaningful limitations of patients’ quality of life. The prevalence of LSS increases with advancing age [1,2] and is expected to be twice as common in adult people older than 65 years [3]. In Europe, with an incidence of 2.2%, 102 million people are thought to be affected annually [4]. Multifactorial degeneration of the lumbar spine resulting in narrowing of the neuroforamina and thus compression of the spinal nerves, leads to symptomatic reduction in walking distance and neurogenic claudication in patients affected by LSS [5].

Therapeutic options depend on the patient's symptoms and concomitant pathologies. However, conservative therapy attempts should be performed first, consisting of physiotherapy, pain therapies, and epidural infiltrations [6]. In case of recurrent symptoms over a period of three to six months after starting conservative treatment, surgical therapy, was found to be superior to continuing conservative therapy only [7]. Available surgical procedures include microsurgical decompression alone, and decompression with fusion using an internal fixator and intervertebral or posterolateral fusion [8]. In post-operative pain and disability, laminectomy with fusion seems to be superior to laminectomy alone [9].

The evaluation of therapy success focuses, among other things, on the progress of walking distance. Alongside pain, walking distance is the leading functional outcome in patients with LSS. However, walking ability and its descriptors used to characterize LSS patients are not homogeneous [10]. Specifically, LSS patients show reduced walking distance, gait velocity and lower extremity strength. Moreover, after a provocation walk (400 feet, approximately 122 m) patients with LSS showed decreased hip flexor and knee extensor strength, which was no longer evident after surgical decompression [11]. Those outcomes were recently found to be moderately to strongly associated with patients' handgrip strength [12]. Thus, handgrip strength seems a promising, easy to record, surrogate measure for functional ability in patients with LSS, especially since radiological findings failed to predict patients’ walking distance [13].

Functional electrophysiological examinations (surface EMG) of muscles in LSS patients are very rare. However, these can provide concrete evidence of muscular function and coordination and thus detect dysfunction or dyscoordination. A correlational study examining gait and muscle activation characteristics could demonstrate increased paraspinal and gluteal muscle activity in patients with LSS [14]. After decompression surgery, patients showed decreased paraspinal and increased gluteal muscle activation [15]. Unfortunately, co-activity or symmetry were not examined here.

Active rehabilitation one to two months after surgery results in pain reduction and improves overall health in patients with lumbar disk degeneration [16]. However, it remains unclear whether LSS patients would benefit from early postoperative rehabilitation, or if a delayed rehabilitation would be more advantageous. In our regional setting, depending on the preference of the surgeon, both approaches are implemented.

This controlled and partially randomized patient preference trial aims to evaluate the impact of post-operative rehabilitation timing as well as surgical intervention type on psychometric properties and functional outcomes in patients with LSS. To identify predictors for intervention success or failure, patient-related outcome measures, functional tests, and surface EMG measurements will be used.

## 2 Methods

### 2.1 Study design

**Fig. 1:**
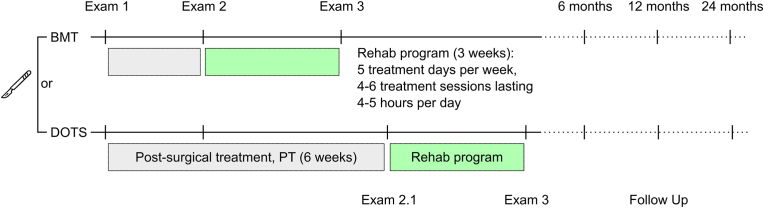
The study flow diagram highlighting the measurement time points (exams) and early and later post-operative rehabilitation intervention groups (BMT, Bergmannstrost. DOTS, Department of Orthopedic and Trauma Surgery. PT, Physiotherapy). Note the conservatively treated group is not considered.

The present study is a partially randomized patient preference trial performed on patients from two different local hospitals. Measurements will be performed before the surgical intervention, at the time when the patient starts standardized rehabilitation, directly after rehabilitation and six, 12 and 24 months after rehabilitation (Fig. 1). For comparative purposes the standardized rehabilitation will be implemented by one local rehabilitation center with similar protocols for the early and late rehabilitation groups. Additionally, a conservatively treated group will be recruited within the rehab center responsible. The study was registered with the German Clinical Trials Register (ID: DRKS00032248). The SPIRIT reporting guidelines were used for this trial protocol [17]. The baseline data collection started in August 2023.

### 2.2 Study participants

The study participants are at least 18 years old. An overview of the inclusion and exclusion criteria is given in Table 1. Patients suffering from LSS will undergo surgical intervention or conservative treatment with a standardized rehabilitation program at the same rehabilitation center for three weeks (15 intervention sessions each lasting 4 to 5 h). LSS patients will be recruited and treated in two local but independent spinal surgery centers. The conservatively treated group will be enrolled at the rehab center. Patients meeting the inclusion criteria listed in Table 1 will be included consecutively in the study. Control participants will be recruited through public announcements. Furthermore, patients will be encouraged to recruit controls without any known lumbar spinal impairments of approximately the same age, sex, and activity status from their own environment.

**Table 1:** Inclusion and exclusion criteria for the patients.

Inclusion criteria	Exclusion criteria
- Age: at least 18 years old - Voluntary participation - Signed consent - Anatomical lumbar spinal stenosis with characteristic symptoms - Present pain (NRS >3) - Agreement to be treated in the selected rehabilitation facility	- Children and adolescents - Neurodegenerative diseases - Previous spine surgery - Fractures on the lumbar spine (<2 years) - Degenerative lumbar scoliosis (>20°) - Spondylolisthesis > Meyerding 1 (3–14 mm) - ASA classification [18] >4 - Pain (NRS <3) - Orthopedic history at the lower extremities, limiting the patient's activity

For representative sampling, the study collective is self-monitored with regard to the proportion of females included, the preferred post-operative rehabilitation timing, and other parameters that may need to be balanced in periodic meetings of the participating study centers.

### 2.3 Assessment procedures

After meeting the inclusion criteria, a brief standard explanation of the aim of the study and the assessments that will be used, participants will be asked to sign the consent form. After completing the medical check-up, which includes demographic information, medical history, anthropometric measurements as well as clinical examinations on a standardized observation sheet, each participant will complete patient-related questionnaires in the same order.

#### 2.3.1 Patient-reported outcome measures

To detect the impact of individual motivation, expectations of rehabilitation and the risk of early retirement, assuming that they have an influence on patient outcome, the following questionnaires will be used: FREM-8, PAREMO-20 and SIBAR. These questionnaires are used in part because of the possibility that patients who are still working may have a hidden desire for early retirement. Patients’ attitude, expectation and goals can, of course, have an impact on the results of rehabilitation.

To detect patients' rehabilitation-related expectation, the FREM-8 will be used. The FREM-8 evaluates in four subscales (expectations about recovery, health, coping with illness, and retirement) on a 4-point graded response scale (0 = not at all true, 3 = exactly true) patients' expectations [19]. The FREM-8 showed an internal consistency of Cronbach's alpha between 0.48 in the health subscale to 0.85 in the retirement subscale [19].

The Patient Questionnaire for Assessment of Rehabilitation Motivation (PAREMO-20) records in six subscales the mental distress, physical limitations, social support and gain from illness, willingness to change, level of information regarding rehabilitation measures, and skepticism [20]. The scales are formed as a sum score of the four level item values (1 = not true, 4 = true). Satisfactory to good internal consistencies (Cronbach's alpha) between 0.59 and 0.88 for skepticism and mental distress were found for the PAREMO-20, respectively [21].

The patient-reported functional outcome and its change during the observation period will be assessed using the well-established 12-Item Short-Form Health Survey (SF-12 [22]) with its mental (MCS) and physical component summary (PCS) subscales, the Oswestry Disability Index (ODI [23,24]) and the Spine Functional Index (SFI [25]). Patients with LSS do not only suffer from back pain. Other disabilities and age can lead to falsification of various items on the established questionnaires such as SF-12 or ODI. For this reason, we try to uncover a better picture of the state of health and the limitations by adding the above-mentioned questionnaires on expectation and motivation and the SFI.

The SF-12 consists of 12 items with responses of two to six-level categories. At 0.77 and 0.80 for the PCS and MCS, respectively, Cronbach's alpha showed good internal consistency [26].

The ODI consists of 10 items with answer options of 5 levels. It detects back pain related functional disability in daily life and was shown to have an overall Cronbach's alpha of 0.90 [24]. However, the greatest functional limitation in LSS patients is the limitation precisely in walking. Also, we expect that because of the anticipated older cohort, the answer to the question of sexual activity might not always be answered adequately.

The SFI is a newer spine-specific questionnaire that overcomes the limitations of existing whole-spine questionnaires and identifies clinical and practical characteristics along with a recognized criterion, the Functional Rating Index (FRI [27]). It showed internal consistency of 0.91 and a high correlation with the FRI of 0.87 [25].

Additionally, the pain medication and the dosage will be recorded according to the WHO grading scheme.

**Fig. 2:**
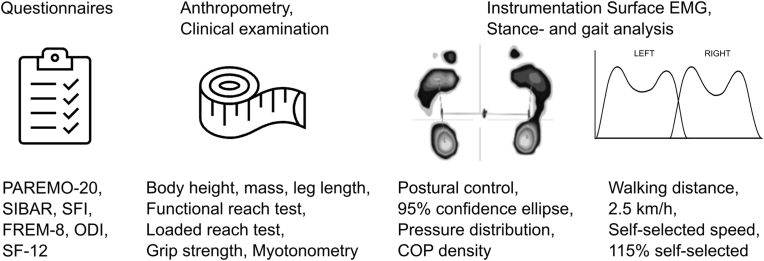
Overview of the assessment procedures (PAREMO-20, Patient Questionnaire for Assessment of Rehabilitation Motivation. SIBAR, Screening Instrument for the subjective need for Occupation related Rehabilitation. SFI, Spine Functional Index. FREM-8, Questionnaire for assessing Rehabilitation related Expectations and Motivations. ODI, Oswestry Disability Index. SF-12, 12-Item Short-Form Health Survey. EMG, Electromyography. COP, Center of pressure).

Before functional, balance and gait analyses, patients’ heart rate variability (HRV) will be examined while lying prone to record autonomic nerve activity. After 5 min rest or relaxation time, 5 min of continuous HRV recording will be performed [28]. This will be followed by measurements of the lumbar resting myofascial tone [[29], [30], [31]] with a portable device (MyotonPRO, Myoton AS, Tallinn, Estonia) applied paravertebral at both sides to detect changes in muscle stiffness during the observation period. Thereafter, the following functional assessments will be completed by each participant: functional reach test (FRT), loaded reach test (LRT), handgrip strength, standing balance control (60s in natural position), and gait analysis on an instrumented treadmill (h/p/cosmos sports & medical gmbh, Nussdorf-Traunstein, Germany) with an integrated pressure measuring platform (FDM-THQ-i3, zebris Medical GmbH, Isny, Germany). Patients will be asked to walk at A) 2.5 km/h for approximately 30s, B) self-selected speed as long as possible or up to 6 min (6-min walk test [[32], [33], [34]]) and, after a sufficient break, C) 115% of the self-selected speed again for approximately 30s. Following each walking task, as well as after each functional test performed, patients will be asked to indicate their individual exertion (rate o perceived exertion, RPE [35]) on an 11-point NRS [36]. For an overview of the assessment procedures used in the study, please refer to Fig. 2. The ODI together with the distance walked at self-selected speed were selected as primary outcome measures, whereas strength (handgrip strength) and functional mobility (FRT) served as secondary outcomes. The experimental outcome categories with exploratory variables are listed in Table 2.

**Table 2:** Experimental outcome categories with exploratory variables.

Outcome categories	Explanatory variables	Unit or range
Resting heart function	Heart rate	bpm
	Heart rate variability (RMSSD)	ms

Resting muscle tone	Oscillation frequency	Hz
	Dynamic stiffness	N/m
	Logarithmic decrement	–
	Mechanical stress relaxation time	ms
	Ratio of relaxation and deformation time	–

Reach distance	Unloaded distance	cm
	Loaded distance	cm

Handgrip strength	Absolute strength	kg
	Relative strength	kg/kg

Standing balance control	COP path length	mm
	COP average velocity	mm/s
	95% confidence ellipse area	mm^2^
	Forefoot average force L	%
	Total average force L	%
	Muscle activation coefficient of variability	%
	Muscle activation symmetry	%
	Glutaeus medius coactivation	0–1

Gait control	Gait velocity (self-selected)	km/h
	Stride length	cm
	Step length L	cm
	Step length R	cm
	Step width	cm
	Stride time	s
	Step time L	s
	Step time R	s
	Cadence	steps/min
	Heel maximum force L	N
	Heel maximum force R	N
	Heel maximum force symmetry	%
	Muscle activation coefficient of variability	%
	Muscle activation symmetry	%

RMSSD, Root Mean Square of Successive Differences. COP, Center of pressure. L, Left. R, Right.

#### 2.3.2 Functional reach test (FRT)

The unloaded FRT quantifies participants' dynamic in-place standing balance control as reach distance. The participant is standing with feet hip-width next to a wall (without touching the wall) with the right arm extended and parallel to the floor. A tape is mounted on the wall at the participant's shoulder height. The starting point is marked before the patient performs symmetrical hip flexion by moving the trunk forward without moving the feet off the ground, bending the knees, and losing balance, i.e., stepping. The distance between the starting and maximal forward reach distance beyond the participant's arm length represents the reach distance [37] and is recorded in centimeters. After two familiarization trials, three measurements will be documented. The best of three attempts will be used for further analyses. The test-retest reliability of the FRT has been found to be good to excellent with intraclass correlation coefficient (ICC) values above 0.80 [37,38]. Despite the fact that participants are required to maintain a neutral trunk position, spinal mobility has been shown to significantly affect the reach distance [39].

#### 2.3.3 Loaded reach test (LRT)

The LRT was inspired by the FRT and controlled spinal loading experiments on patients with low back pain [40]. It basically aims to load the posterior chain while extending both arms and leaning forward. The participant holds an additional weight with both hands. Depending on the participant's body mass, weights with two (<70 kg), three (60–80 kg) or four (>80 kg) kilograms are used [41]. The reach distance is determined according to the FRT. After two familiarization trials, three measurements will be recorded. The test-retest and inter-rater reliability of the LRT has been found to be excellent in participants with and without low back pain [42].

#### 2.3.4 Handgrip strength measurement

Handgrip strength will be measured seated at patients’ dominant side using a handheld dynamometer (SH5001, Saehan Corporation, Masan, South Korea). The position of the upper extremity is with the shoulder adducted and neutrally rotated, elbow flexed at 90° and forearm in neutral. Two maximum attempts will be recorded after two submaximal familiarization trials. For all participants, the second handle position will be used, as this is supposed to be the most reliable and consistent grip widths in adults [43]. The test-retest reliability of the handgrip strength testing in young adults during sitting has been found to be excellent, with ICC values above 0.9 [44].

#### 2.3.5 Follow-up

Follow-up assessments will be performed at six, 12 and 24 months after completing the rehabilitation program.

#### 2.3.6 Power analysis and sample size considerations

The ODI was set as our primary outcome measure. For this study, we approximated the sample size using a hypothetical approach. In order to estimate the number of cases needed, the ODI results of 46 patients with LSS (41% men) who underwent fusion surgery were used [45]. Considering the mean change from baseline of 16.1 points and the standard deviation of 17.8, a large effect (d = 0.9) can be expected. With a significance level of 5% and a power of 95%, the minimum sample size needed with this effect size is 15 patients with LSS who will undergo fusion surgery [one-sample *t*-test] (G*Power 3.1.9.2). Only 65% (30) of the patients reduced their ODI by at least ten points one year post-operatively [45]. Taking into account that approximately two-third of the patients included will reach the minimum clinically important difference and assuming a relatively high dropout rate of 20%, 30 patients with LSS would have to be initially included in each group.

#### 2.3.7 Statistical analyses

The data collected will be used to answer various research questions. Hence different analyses will be conducted. First, data quality checking will be performed to ensure that discrepancies, errors, or duplicates are excluded from further analyses and overall data consistency is given. Second, data will undergo exploratory data analysis. Finally, depending on the variables available, appropriate statistical tests (e.g., linear regressions, Student's t-tests, Chi^2^-tests) will be applied to uncover differences related with rehabilitation timing after LSS surgery. Statistical analyses will be performed using IBM SPSS 28.0 (IBM Corp., Armonk, NY, USA) software.

a) Subjective (ODI, SF-12) and functional performance (handgrip strength, FRT) of early and later post-operative rehabilitation intervention groups will be compared using multivariate general linear mixed model and Chi^2^-test (categorical data, specifying frequencies in percent).
b) Subjective and functional performance of surgically (two groups) and conservatively treated patients with LSS and controls without spinal impairments will be compared using multivariate general linear mixed model and Chi^2^-test (categorical data, specifying frequencies in percent).
c) Subjective and functional performance before and after the intervention(s) will be compared using multivariate general linear mixed model and Chi^2^-test (categorical data, specifying frequencies in percent).
d) Associations of subjective and functional assessments will be tested by means of correlation analyses.

#### 2.3.8 Patient and public involvement (PPI) statement

Early or delayed start of the rehabilitation program will be randomized for the surgically treated patients. If patients don't want to be randomized, they can choose their preferred starting time of the post-surgical rehabilitation program. Control participants will be recruited through public announcements. Furthermore, patients will be encouraged to recruit controls without any known lumbar spinal impairments from their own environment in order to increase the chance of getting comparable control subjects in terms of sex, age, and activity level. Patients' and controls' will be asked about their experience with the assessments in order to better estimate and compare the exhibited strain and perceived exertion of the functional tests.

## 3 Ethics and dissemination

The present study has been approved by the Institutional Review Board of Martin Luther University Halle-Wittenberg (reference number: 2022-128) and Ärztekammer Sachsen-Anhalt (reference number 23/34). Prior to enrolment in the study, all patients will be asked to give their written informed consent. The patient can decide at any time to be released from the study, and they will be made aware of this in the information leaflet. Their data will then be deleted from the data collection file. Voluntary termination of study participation will have no disadvantages for the patients.

## 4 Results

The research results from this study will be spread through peer-reviewed publications and scientific contributions at national and international conferences.

## Ethical approval and consent to participate

Institutional Review Board approval was obtained through the Ethics Committee of Martin Luther University Halle-Wittenberg (reference number: 2022-128). Significant changes to the protocol will be submitted as an amendment to the Ethics Committee and updated in the trial registry. All patients will be asked to give their written informed consent. All methods will be carried out in accordance with relevant guidelines and regulations.

## Consent for publication

Not applicable.

## Availability of data and materials

The datasets collected in this study will be available from the corresponding author on reasonable request.

## Funding

This work is currently unsupported by external funding sources. The institutions involved will primarily use budgetary resources. External funding options will be considered.

## CRediT authorship contribution statement

**Eduard Kurz:** Conceptualization, Investigation, Methodology, Software, Validation, Visualization, Writing – original draft, Writing – review & editing. **Philipp Schenk:** Conceptualization, Investigation, Methodology, Software, Validation, Visualization, Writing – original draft. **Florian Brakopp:** Investigation, Writing – original draft. **Moritz Diers:** Investigation, Writing – original draft. **Oliver Klingel:** Conceptualization, Investigation, Writing – original draft. **Stefan Bone:** Conceptualization, Investigation, Writing – original draft. **Hans Jörg Meisel:** Conceptualization, Funding acquisition, Methodology, Project administration, Resources, Supervision, Writing – original draft. **Karl-Stefan Delank:** Conceptualization, Funding acquisition, Methodology, Project administration, Resources, Supervision, Writing – original draft. **Bernhard W. Ullrich:** Conceptualization, Funding acquisition, Methodology, Project administration, Resources, Supervision, Writing – original draft.

## Declaration of competing interest

The authors declare that they have no known competing financial interests or personal relationships that could have appeared to influence the work reported in this paper.
